# Description of new species of *Stenaelurillus* Simon, 1886 from the Western Ghats of India with the redescription of *Stenaelurillus lesserti* Reimoser, 1934 and notes on mating plug in the genus (Arachnida, Araneae, Salticidae)

**DOI:** 10.3897/zookeys.491.8218

**Published:** 2015-03-26

**Authors:** Pothalil A. Sebastian, Pradeep M. Sankaran, Jobi J. Malamel, Mathew M. Joseph

**Affiliations:** 1 Division of Arachnology, Department of Zoology, Sacred Heart College, Thevara, Cochin, Kerala 682 013, India

**Keywords:** Mating plug, new species, paternity, redescription, Western Ghats

## Abstract

A new species of the jumping spider genus *Stenaelurillus* Simon, 1886, *Stenaelurillus albus* **sp. n.**, is described from the Western Ghats of India, one of the biodiversity hotspots of the world. Detailed morphological descriptions, diagnostic features and illustrations of copulatory organs of both sexes are given. Detailed redescription, diagnosis and illustration of *Stenaelurillus lesserti* Reimoser, 1934 are provided. The occurrence of a mating plug in the genus is reported.

## Introduction

The salticid spider genus *Stenaelurillus*, which is considered a senior synonym of *Philotheroides* Strand, 1934 (Prószyński 1984), was erected by Simon in 1886 to accommodate three species: *Stenaelurillus nigricaudus* Simon, 1886 (from Senegal), *Stenaelurillus nigritarsus* Simon, 1886 (from Algeria), which later became a junior synonym of *Stenaelurillus nigricaudus* (Szűts & Scharff, 2005) and *Stenaelurillus triguttatus* Simon, 1886 (from Tibet). Wesolowska (2014) reviewed the Asian species of the genus and synonymised *Stenaelurillus hainanensis* Peng, 1995 with *Stenaelurillus minutus* Song & Chai, 1991 and considered *Stenaelurillus setosus* Thorell, 1895 as a *nomen nudum*. Presently the genus has 27 valid species, mostly from Africa (21 species) with only two representatives from India, *Stenaelurillus lesserti* Reimoser, 1934 (known from both sexes) and *Stenaelurillus sarojinae* Caleb & Mathai, 2014 (known only from female) (World Spider Catalog 2014). The current paper provides the description of a new species of the genus *Stenaelurillus* from the Western Ghats, one of the biodiversity hotspots of the world (Myers et al. 2000), in the Kerala region of southern India with the redescription of *Stenaelurillus lesserti* Reimoser, 1934.

## Material and methods

The specimens were preserved in 70% ethanol and studied under a Zeiss Stemi 2000-C stereomicroscope. All measurements are in millimetres (mm) and were made with an ocular micrometer. Length of palp and leg segments are given as: total (femur, patella, tibia, metatarsus (except palp), tarsus). Spine positions are as follows: prolateral, dorsal, retrolateral and ventral. Comparison of the new *Stenaelurillus* species with all other described species is based only on available literature. Drawings were made by the aid of a drawing tube attached to the microscope. Field photos were taken with Canon EOS 6D with Canon Macro photo lens MP-E65 mm 1:2.8 lens attached. The microphotographic images were taken by Leica DFC295 digital camera attached to Leica M205 C stereomicroscope with the software package Leica Application Suite (LAS), version 4.3.0. All specimens are deposited in a reference collection housed at the Division of Arachnology, Department of Zoology, Sacred Heart College, Thevara, Cochin, Kerala, India (ADSH).

### Abbreviations

ALE–anterior lateral eye, AME–anterior median eye, CD–copulatory duct, CO–copulatory opening, E–embolus, PLE–posterior lateral eye, PME–posterior median eye, RPT–retrobasal process of tegulum, RTA–retrolateral tibial apophysis, S–spermatheca, T–tegulum, TA–terminal apophysis, VTA1 & VTA2–ventral tibial apophyses 1 & 2; VPT–ventral process of tegulum, WS–weakly sclerotized part of copulatory duct.

## Taxonomy

### Salticidae Blackwall, 1841
Aelurillinae Simon, 1901

#### Stenaelurillus

Taxon classificationAnimaliaAraneaeSalticidae

Simon, 1886

##### Diagnosis.

Medium sized spiders. Prosoma of all the known *Stenaelurillus* species has two white transverse stripes. Both male and female have strong bristles on the ocular area. Male palp with a short, not coiled and visible embolus, and tegulum with characteristic retrobasal process. RTA is simple and strongly sclerotized. Epigyne is simple, with thick-walled copulatory openings and short copulatory ducts, and is often accompanied by accessory glands (Szűts and Scharff 2005).

##### Type species.

*Stenaelurillus nigricaudus* Simon, 1886, by original designation.

##### Distribution.

Africa, Asia (World Spider Catalog 2014).

#### Stenaelurillus albus sp. n.

Taxon classificationAnimaliaAraneaeSalticidae

http://zoobank.org/29A9E0C0-1472-47A2-A9BB-22B4D84C8C01

**Figure 1.**
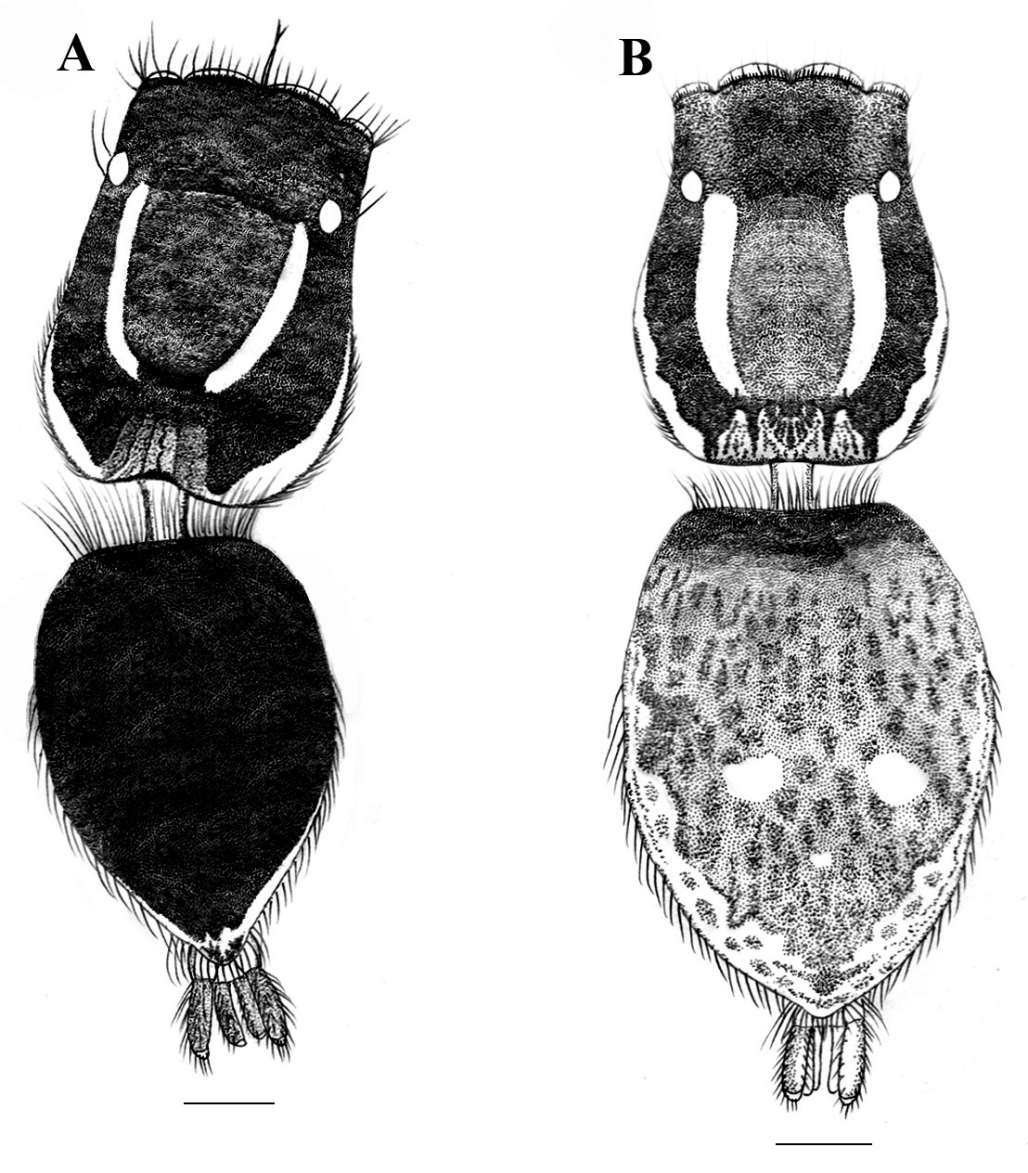
*Stenaelurillus albus* sp. n. **A** Male habitus, dorsal view **B** Female habitus, dorsal view. Scale bars: **A** = 0.58 mm; **B** = 0.68 mm.

**Figure 2.**
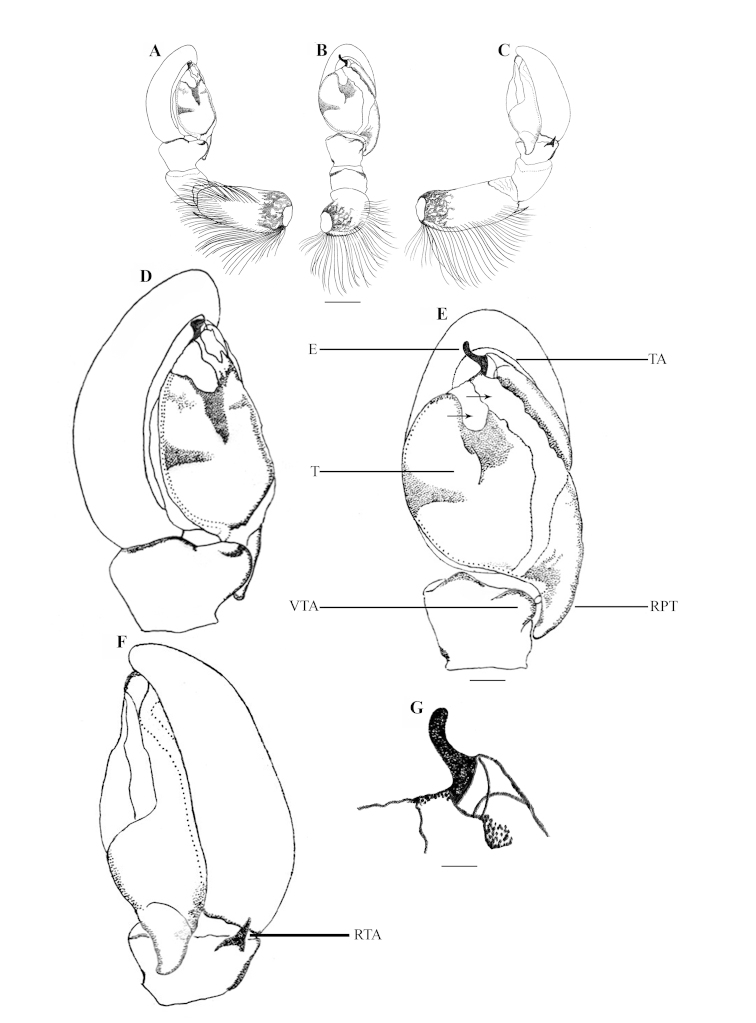
*Stenaelurillus albus* sp. n. Left male palp. **A** Palp entire, prolateral view **B** Same, ventral view **C** Same, retrolateral view **D** Palp enlarged, prolateral view **E** Same, ventral view **F** Same, retrolateral view **G** Embolic division of the bulb, ventral view. E = Embolus; RPT = Retrobasal process of tegulum; RTA = Retrolateral tibial apophysis; T = Tegulum; TA = Terminal apophysis. Scale bars: **A–C** = 0.34 mm; **D–F** = 0.08 mm; **G** = 0.02 mm.

**Figure 3.**
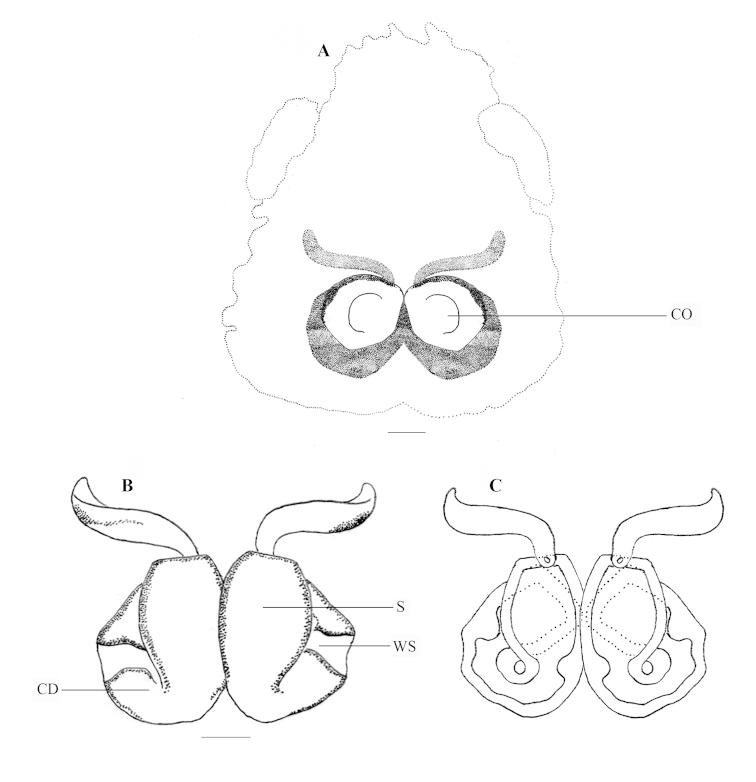
*Stenaelurillus albus* sp. n. Female copulatory organ. **A** Epigyne **B, C** Internal duct system. CO = Copulatory opening; CD = Copulatory duct, S = Spermatheca; WS = Weakly sclerotized part of copulatory duct. Scale bars: **A** = 0.11 mm; **B–C** = 0.03 mm.

Figs 1A–B , 2A–G , 3A–C , 7A , 8A–I , 9A–F

##### Type material.

Holotype: Male (ADSH 83503Ai): India, Kerala, Ernakulam, Kurisumudi (10°12'33.36"N, 76°30'08.85"E) in Malayatoor (10°11'43.76"N, 76°29'48.45"E), 94 m. alt., Pradeep M. S., 04. XII. 2013, by hand; Paratypes: 8 females, 6 males (ADSH 83503Aii), same data as holotype.

##### Diagnosis.

Males of *Stenaelurillus albus* sp. n. can be separated from all other described congeners by uniformly dark dorsal opisthosoma without any pattern (Figs 1A, 8A), paired creamy white areas at the anterior part of the bulbus, (Fig. 2E, arrows) and palpal femur with a single disto-dorsal spine (Figs 2A, 2C); females are most similar to *Stenaelurillus abramovi* Logunov, 2008 as both possess wide copulatory openings (Logunov 2008, Fig. 4 and herein Figs 3A, 9E), but can be distinguished by the presence of small and ‘vase’- shaped spermathecae (Figs 3B, 9F) and copulatory ducts with weakly sclerotized anterior part (Fig. 3B).

##### Description.

MALE (holotype, Figs 1A, 8A–F): Prosoma black with white lateral bands of nearly uniform thickness; thoracic region dorsally with paired white longitudinal bands extending back from the rear eyes. Eye field black; anterior row of eyes encircled by black hairs. Clypeus densely covered with white hairs, which is a continuation with that on the lateral margins of carapace. Chelicerae short, vertical, brownish with a thick mid-dorsal transverse layer of white hairs; promargin with two, one large and one small, and retromargin with one large teeth. Fangs short, pale brown. Maxillae, labium and sternum yellowish-brown. Opisthosoma oval; dorsum uniformly shiny black without any pattern; lateral opisthosoma and venter dull yellow with several broken black striations and spots. Trochanters III and IV and coxae III and IV yellowish-brown; trochanters I and II and coxae I and II black; all tarsi brown; all other leg segments dull yellow with broad black patches giving a blackish appearance to the legs. Body length 5.89. Prosoma length 2.98, width (at the middle) 2.08, height (at the middle) 1.72. Opisthosoma length 2.91, width (at the middle) 1.98, height (at the middle) 1.45. Eye diameter: AME 0.44. ALE 0.22. PME 0.06. PLE 0.23. Eye interdistance: AME–AME 0.04. PME–PME 1.35. PLE–PLE 1.33. AME–ALE 0.06. ALE–PLE 0.41. ALE–PME 0.27. PME–PLE 0.14. Clypeus height at AMEs 0.36, at ALEs 0.64. Chelicera length 0.65. Measurements of palp and legs. Palp 1.78 [0.70, 0.32, 0.16, 0.60], I 4.25 [1.40, 0.81, 0.90, 0.60, 0.54], II 4.14 [1.45, 0.75, 0.84, 0.59, 0.51], III 6.60 [2.02, 1.14, 1.33, 1.51, 0.60], IV 6.26 [1.94, 0.87, 1.28, 1.53, 0.64]. Leg formula: 3412. Spination. Palp. 0100, 0000, 0000, 0000; legs: femur I 1500, II 1520 (right 1510), III 0300 (right 1300), IV 1500; patellae I–IV 1000; tibia I 2000, II 2022, III 2122, IV 2033 (right 2023); metatarsus I 2014, II 2022, III 3523 (right 3423), IV 2423; tarsi I–IV 0000. Copulatory organ (Figs 2A–G, 8G–I): Palpal segments pale yellow, the basal 1/4^th^ of femur with black striations; femur disto-dorsally with a short spine and dorsally and laterally with a fringe of long yellowish-white hairs, dorsal and prolateral ones are prominent; patella and tibia disto-dorsally with a long black hair; patella ventro-laterally covered with short yellowish-white hairs; cymbium dark and dorsally with a few long black hairs. Bulb brown; anterior edge of the bulbus has two creamy-white regions, the distal one runs retrolaterally to near the antero-lateral edge of the ventral tibial apophysis (Fig. 2E, arrows); retro-basal process of tegulum not fused with the tibia; embolus short with blunt end and is prolaterally directed (Figs 2D–E, 2G); conductor apparently absent; terminal apophysis short, directed at eleven o'clock position (Figs 2E, 2G). VTA short with blunt end and directed at two o'clock position (Fig. 2E); RTA simple with broad base and pointed end and directed at one o'clock position (Fig. 2F).

FEMALE (Paratype, Figs 1B, 9A–D): Prosoma black with dull yellow lateral bands, the thoracic part of which is broader; thoracic region dorsally with paired white longitudinal bands extending back from the rear eyes. Eye field black; anterior row of eyes encircled with dull yellow hairs. Clypeus black; Chelicerae short, vertical and dull yellow; promargin with two, one large and one small, and retromargin with one large teeth. Maxillae, labium and sternum yellowish-brown. Opisthosoma widely oval; dorsum black with several dull yellow patches, the posterior three are prominent, which together forming an inverted triangle; lateral opisthosoma and venter dull yellow with several broken black striations and spots. Leg segments dull yellow with black patches and narrow transverse stripes. Palpal segments yellow with black patches; patella, tibia and tarsus dorsally with long black hairs. Body length 6.82. Prosoma length 2.99, width (at the middle) 2.29, height (at the middle) 1.94. Opisthosoma length 3.83, width (at the middle) 2.84, height (at the middle) 2.27. Eye diameter: AME 0.53. ALE 0.24. PME 0.06. PLE 0.21. Eye interdistance: AME–AME 0.05. PME–PME 1.50. PLE–PLE 1.45. AME–ALE 0.07. ALE–PLE 0.50. ALE–PME 0.31. PME–PLE 0.19. Clypeus height at AMEs 0.38; at ALEs 0.41. Chelicera length 0.63. Measurements of palp and legs. Palp 1.99 [0.69, 0.29, 0.33, 0.68], I 4.06 [1.42, 0.72, 0.86, 0.52, 0.54], II 3.76 [1.28, 0.76, 0.77, 0.47, 0.48], III 6.95 [2.18, 1.05, 1.48, 1.65, 0.59], IV 6.78 [1.97, 0.95, 1.39, 1.77, 0.70]. Leg formula: 3412. Spination. Palp 0100, 0000, 0000, 1020; legs: femora I–II 0700, III 2700, IV 0700; patellae I–II 1000, III–IV 1010; tibia I 3004, II 1004, III 4133, IV 4143; metatarsus I 3003, II 3013, III 3234, IV 4054; tarsi I–IV 0000. Copulatory organ (Figs 3A–C, 9E–F): Spermathecae small with a characteristic vase- shape (Figs 3B, 9F). Copulatory opening wide (Figs 3A, 9E) and nearly half the size of the spermathecae. Anterior part of copulatory duct near the copulatory opening is weakly sclerotized (Fig. 3B).

##### Variation.

Male: Body length 4.61–5.89 (n = 7). Female: Body length 5.43–6.82 (n = 8).

##### Etymology.

The specific epithet is an adjective and is derived from the whitish part of the tegulum: Latin *Albus* = white. Gender musculine.

##### Habitat.

Rocky area covered with litter in a deciduous forest (Fig. 12A).

##### Distribution.

At present known only from the type locality.

#### Stenaelurillus lesserti

Taxon classificationAnimaliaAraneaeSalticidae

Reimoser, 1934

**Figure 4.**
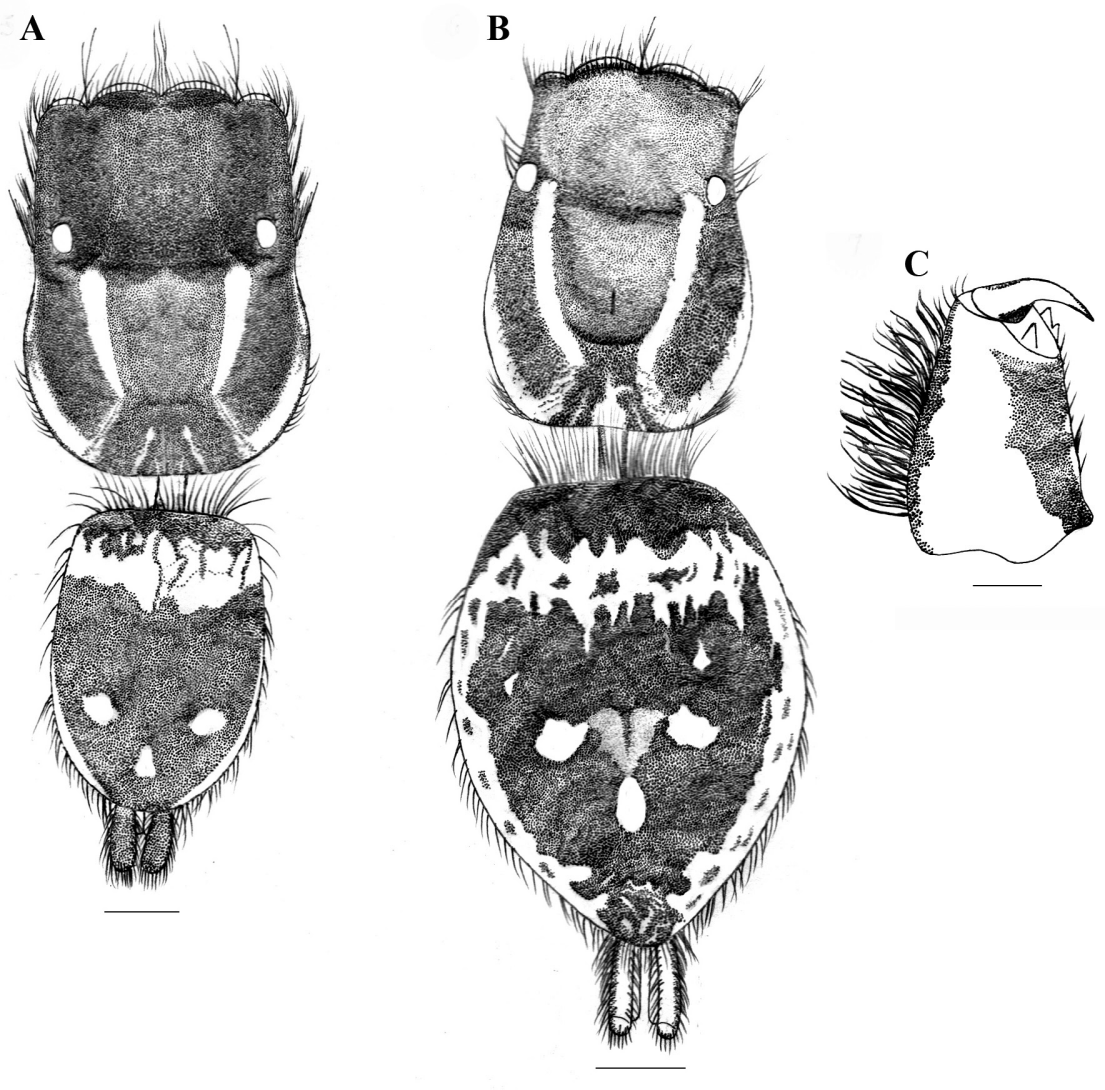
*Stenaelurillus lesserti* Reimoser, 1934. **A** Male habitus, dorsal view **B** Female habitus, dorsal view **C** Male right chelicera, retrolateral view. Scale bars: **A** = 0.38 mm; **B** = 0.65 mm; **C** = 0.08 mm.

**Figure 5.**
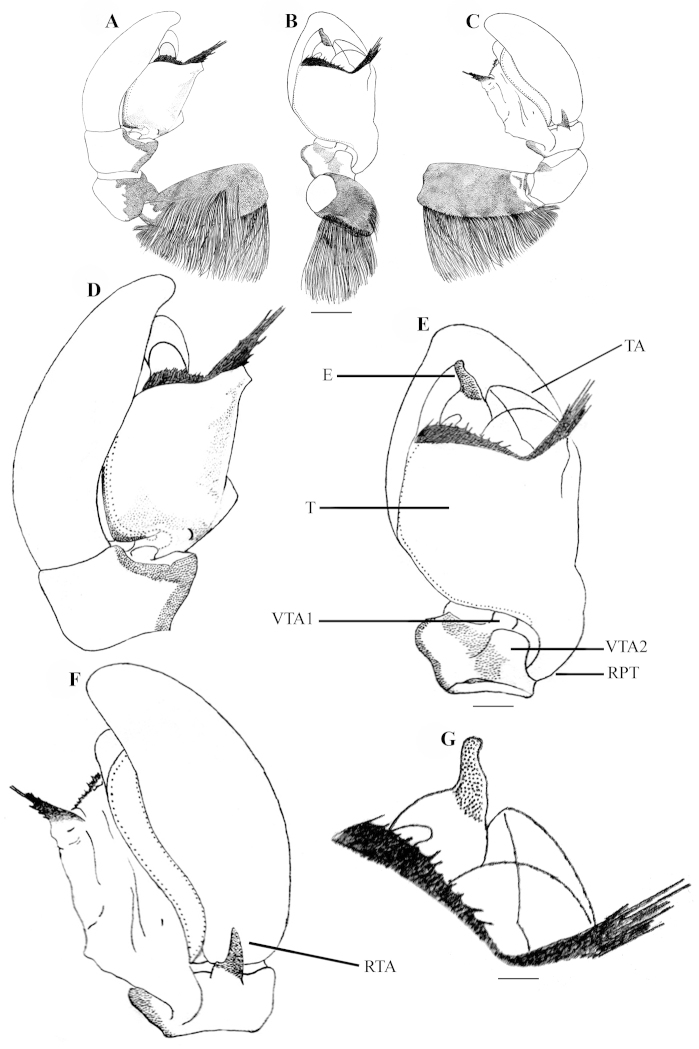
*Stenaelurillus lesserti* Reimoser, 1934. Left male palp. **A** Palp entire, prolateral view **B** Same, ventral view **C** Same, retrolateral view **D** Palp enlarged, prolateral view **E** Same, ventral view **F** Same, retrolateral view **G** Embolic division of the bulb, ventral view. E = Embolus; RPT = Retrobasal process of tegulum; RTA = Retrolateral tibial apophysis, T = Tegulum; TA = Terminal apophysis; VTA 1 & 2 = Ventral tibial apophyses 1 & 2. Scale bars: **A–C** = 0.26 mm; **D–F** = 0.08 mm; **G** = 0.02 mm.

**Figure 6.**
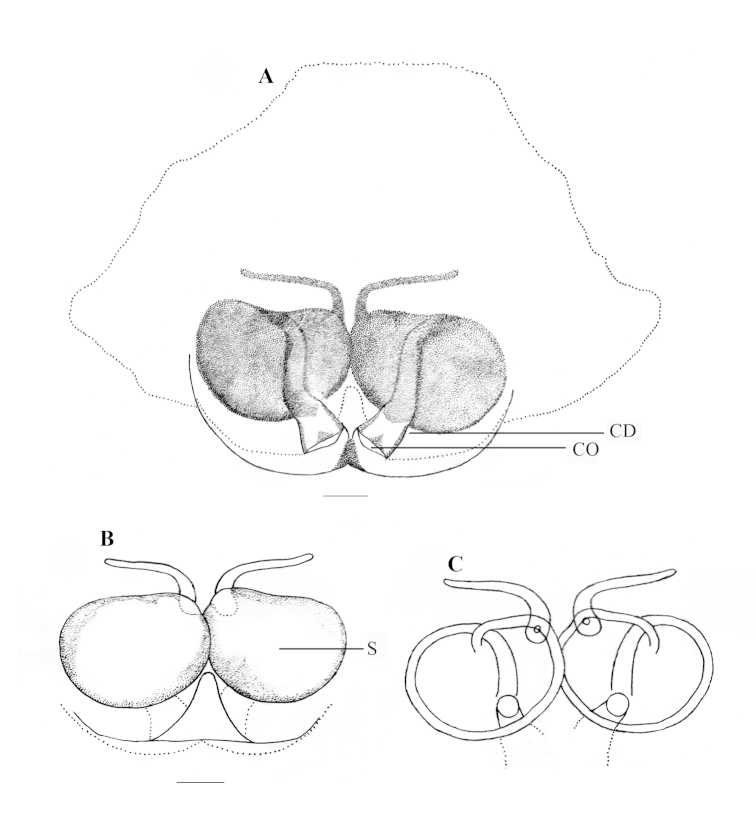
*Stenaelurillus lesserti* Reimoser, 1934. Female copulatory organ. **A** Epigyne **B, C** Internal duct system. CO = Copulatory opening; CD = Copulatory duct; S = Spermatheca. Scale bars: **A** = 0.09 mm; **B–C** = 0.07 mm.

**Figure 7.**
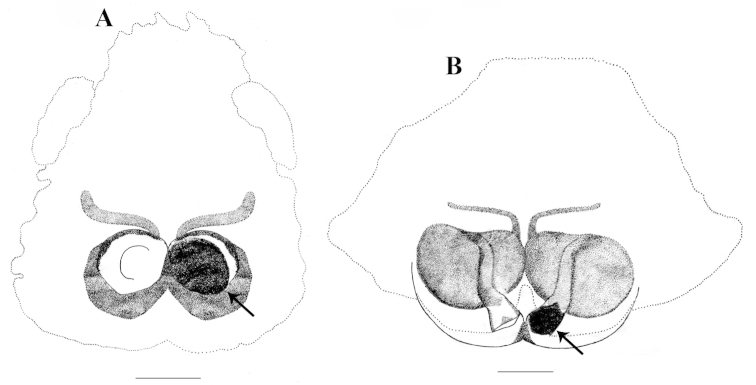
*Stenaelurillus* spp. Epigyne with mating plugs. **A** Epigyne of *Stenaelurillus albus* sp. n. showing mating plug (arrow) **B** Epigyne of *Stenaelurillus lesserti* Reimoser, 1934 showing mating plug (arrow). Scale bars: **A** = 0.21 mm; **B** = 0.19 mm.

Figs 4A–C , 5A–G , 6A–C , 7B , 10A–J , 11A–F

Stenaelurillus lesserti Reimoser, 1934: 504, figs 25–26 (Description and illustration of ♂ and ♀); Prószyński 1984: 139 (Illustration of ♀); Wesolowska 2014: 248, figs 1A–B, 2A–F, 3A–D (Re-examined the original type series of *Stenaelurillus lesserti*; description and illustration of ♂ and ♀).

##### Material examined.

(ADSH 83503Ai)–4 males, 5 females: India, Kerala, Ernakulam, Cherukadu (10°08'22.48"N, 76°40'02.14"E) in Bhoothathankettu Forest Reserve (10°08'22.79"N, 76°40'02.09"E), 37 m. alt., Pradeep M. S., 10. X. 2013, by hand.

##### Diagnosis.

Males of *Stenaelurillus lesserti* Reimoser, 1934 can be separated from all other described congeners by a transverse fringe of very thin, hard projections resembling hairs at the anterior edge of the harder shield covering the bulbus (Figs 5D–G, 10H–J); females by the presence of unusually enlarged and kidney-shaped spermathecae and the relative position of the copulatory openings (Figs 6A–B, 11E–F).

##### Redescription.

MALE (Figs 4A, 4C, 10A–G): Prosoma black, thoracic part with broad yellowish-white lateral bands; thoracic region dorsally with paired white longitudinal bands extending back from the rear eyes. Eye field black with covering of violet scales; anterior row of eyes encircled with red and yellow scales and black hairs. Clypeus covered with transverse layers of orange-red, black and green scales and two layers of greyish-black and red hairs. Chelicerae short, yellowish-brown; dorso-laterally covered with thick layer of red, yellow and green hairs; promargin with two, one large and one small, and retromargin with one large teeth (Fig. 4C). Fangs short, yellow. Maxillae and labium black. Opisthosoma U–shaped; dorsum black with an anterior broad transverse white band and posterior three white spots, which together forming an inverted triangle; lateral opisthosoma pale yellow with several broken longitudinal black striations, while venter pale yellow without any striations or spots. Sternum and coxae pale yellow; coxa I retrolaterally black; femur I pro and retrolaterally black with a prolateral red stripe at the middle (Fig. 10G); femur I prolaterally and dorsally provided with a fringe of black hairs, the dorsal one prominent; ventrally with a row of short white hairs; all other leg segments pale yellow with black patches and narrow transverse black stripes. Body length 3.80. Prosoma length 2.08, width (at the middle) 1.47, height (at the middle) 1.27. Opisthosoma length 1.72, width (at the middle) 1.15, height (at the middle) 0.99. Eye diameter: AME 0.34. ALE 0.19. PME 0.04. PLE 0.18. Eye interdistance: AME–AME 0.04. PME–PME 1.15. PLE–PLE 1.07. AME–ALE 0.07. ALE–PLE 0.45. ALE–PME 0.28. PME–PLE 0.17. Clypeus height at AMEs 0.22, at ALEs 0.42. Chelicera length 0.31. Measurements of palp and legs. Palp 1.44 [0.53, 0.19, 0.16, 0.56], I 3.2 [1.07, 0.52, 0.69, 0.44, 0.48], II 2.91 [0.99, 0.45, 0.59, 0.43, 0.45], III 4.68 [1.45, 0.70, 0.94, 1.03, 0.56] IV 4.48 [1.25, 0.75, 0.76, 1.22, 0.50]. Leg formula: 3412. Spination. Palp. 0000 0000 0000 0000; legs: femur I 0700, II 0710, III 0700, IV 0600, patellae I–II 1000, III–IV 1010; tibia I 3004, II 3014, III 3143, IV 4143; metatarsus I 2014, II 2024, III 4043, IV 5062; tarsi I–IV 0000. Copulatory organ (Figs 5A–G, 10H–J): Femur black, provided prolaterally and dorsally with a bunch of black hairs (Figs 5A–C, 10H–J), the dorsal one is prominent. Patella and tibia pale yellow with ventral black patch. Cymbium and bulb pale yellow; the anterior edge of the harder shield covering the bulbus is provided with a transverse fringe of very thin, hard projections resembling hairs, which are distinctly longer at the retrolateral angle (Figs 5D–F); embolus short with blunt end, retrolaterally directed with prolaterally directed tip (Figs 5E, 5G); conductor apparently absent; terminal apophysis short, directed at ten o'clock position (Figs 5E, 5G). Tibia with two ventral apophyses (Fig. 5E); VTA 1 is the smallest; VTA 2 with a flattened end (Fig. 5E); both VTA 1 & 2 directed at one o'clock position (Fig. 5E); RTA simple with broad base, pointed end, directed at eleven o'clock position (Fig. 5F).

FEMALE (Figs 4B, 11A–D): Prosoma black, thoracic part with broad yellowish-white lateral bands; thoracic region dorsally with paired white longitudinal bands extending back from the rear eyes. Eye field black; anterior row of eyes encircled by pale yellow hairs. Clypeus black. Chelicerae short and black; promargin with two, one large and one small, and retromargin with one large teeth. Fangs short, black. Maxillae and labium black. Opisthosoma widely oval; dorsum black with an anterior broad transverse white band and posterior three white spots, which together forming an inverted triangle; lateral opisthosoma pale yellow with several broken longitudinal black striations while venter pale yellow without any striations or spots. Sternum and coxae pale yellow; coxa I retrolaterally black; all other leg segments pale yellow with black patches and narrow transverse black stripes. Palp: posterior 1/4^th^ of femur black; rest of femur and other segments pale yellow. Body length 6.46. Prosoma length 2.97, width (at the middle) 1.86, height (at the middle) 1.49. Opisthosoma length 3.49, width (at the middle) 2.69, height (at the middle) 1.90. Eyes diameter: AME 0.41. ALE 0.22. PME 0.04. PLE 0.15. Eye interdistance: AME–AME 0.02. PME–PME 1.31. PLE–PLE 1.14. AME–ALE 0.05. ALE–PLE 0.38. ALE–PME 0.21. PME–PLE 0.16. Clypeus height at AMEs 0.27, at ALEs 0.44. Chelicera length 0.56. Measurements of palp and legs. Palp 1.5 [0.52, 0.25, 0.24, 0.49], I 3.28 [1.14, 0.58, 0.61, 0.44, 0.51], II 3.16 [1.12, 0.54, 0.58, 0.49, 0.43], III 5.49 [1.75, 0.84, 1.09, 1.14, 0.67], IV 5.13 [1.53, 0.66, 1.07, 1.26, 0.61]. Leg formula: 3412. Spination. Palp. 0100 0000 0000 2231; legs: femur I 0600, II 0700, III 2600, IV 0600; patellae I–II 1000, III–IV 1010; tibia I 1005, II 2004, III 4043, IV 4343; metatarsi I–II 2014, III 3324, IV 3323; tarsi I–IV 0000. Copulatory organ (Figs 6A–C, 11E–F): Copulatory opening is nearly diamond shaped (Fig. 6A) and placed near the posterior margin of the epigyne (Figs 6A, 11E). Spermathecae are much enlarged and kidney shaped (Figs 6B, 11F).

##### Variation.

Male: (n = 4) Body length 3.24–3.80. Female: (n = 5) Body length 6.15–6.46.

##### Habitat.

Rocky area having patches of grass and herbaceous vegetation in a semi-evergreen forest (Fig. 12B).

##### Distribution.

India, Sri Lanka (Wesolowska 2014).

##### Distribution in India.

Kerala (new record) and Tamilnadu (Reimoser 1934).

##### Note.

Mating plugs, which are supposed to function as paternity protection devices (Uhl et al. 2010; Herberstein et al. 2012), are not very unusual in the animal kingdom and their presence have been described in a number of spider families including Salticidae. Mating plugs are reported in a total of 10 genera and 17 species of salticid spiders (Uhl et al. 2010). Mating plug was observed in the copulatory opening of the two *Stenaelurillus* species described in this paper. The left copulatory opening of both *Stenaelurillus albus* sp. n. and *Stenaelurillus lesserti* were found to be sealed with amorphous secretions (whether male or female origin is unclear) (Figs 7A–B, arrows). Compared to *Stenaelurillus lesserti*, the mating plug of *Stenaelurillus albus* sp. n. is more prominent and covering nearly the whole area of the left copulatory opening and the surrounding epigynal region.

**Figure 8.**
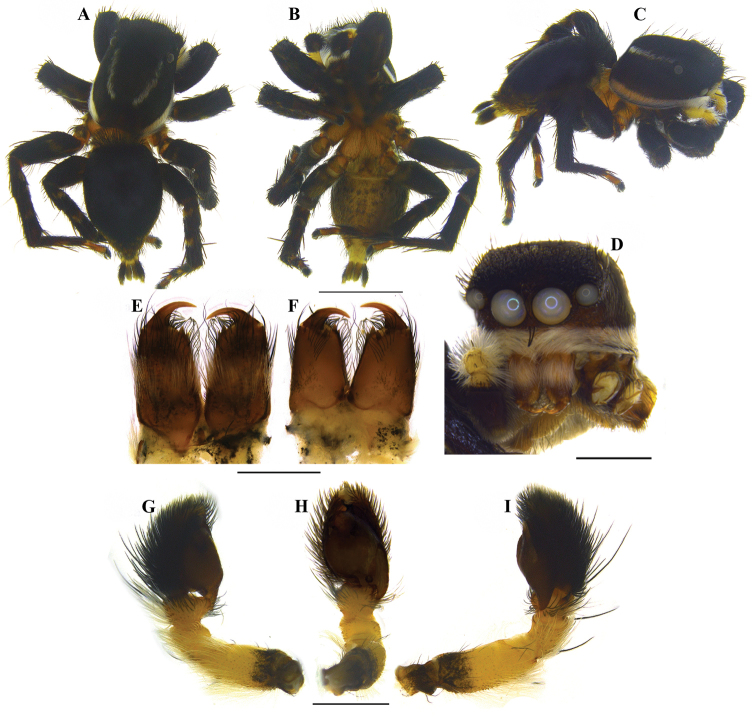
*Stenaelurillus albus* sp. n. **A** Male habitus, dorsal view **B** Same, ventral view **C** Same, prolateral view **D** Same, frontal view **E** Male chelicerae, dorsal view **F** Same, ventral view **G** Left male palp, prolateral view **H** Same, ventral view **I** Same, retrolateral view. Scale bars: **A–C** = 2 mm; **D** = 1 mm; **E–F** = 0.5 mm; **G–I** = 0.5 mm.

**Figure 9.**
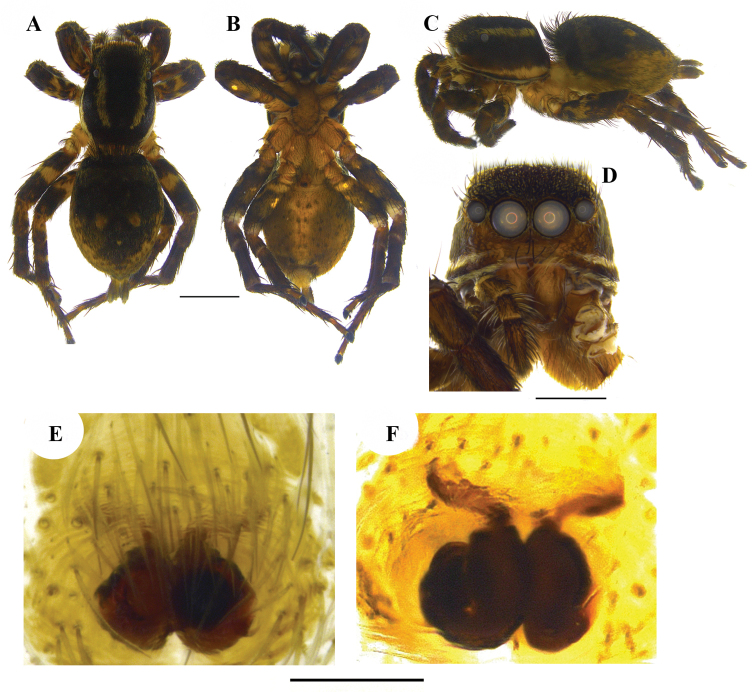
*Stenaelurillus albus* sp. n. **A** Female habitus, dorsal view **B** Same, ventral view **C** Same, retrolateral view **D** Same, frontal view **E** Epigyne **F** Internal duct system. Scale bars: **A–C** = 2 mm; **D** = 1 mm; **E–F** = 0.2 mm.

**Figure 10.**
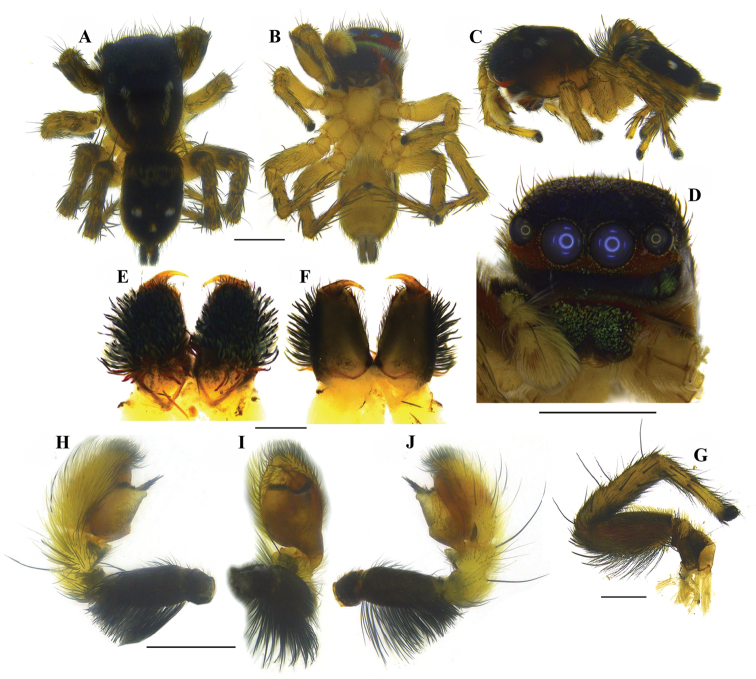
*Stenaelurillus lesserti* Reimoser, 1934. **A** Male habitus, dorsal view **B** Same, ventral view **C** Same, retrolateral view **D** Same, frontal view **E** Male chelicerae, dorsal view **F** Same, ventral view **G** Male left leg I, prolateral view **H** Left male palp, prolateral view **I** Same, ventral view **J** Same, retrolateral view. Scale bars: **A–C** = 1 mm; **D** = 1 mm; **E–F** = 0.2 mm; **G** = 0.5 mm; **H–J** = 0.5 mm.

**Figure 11.**
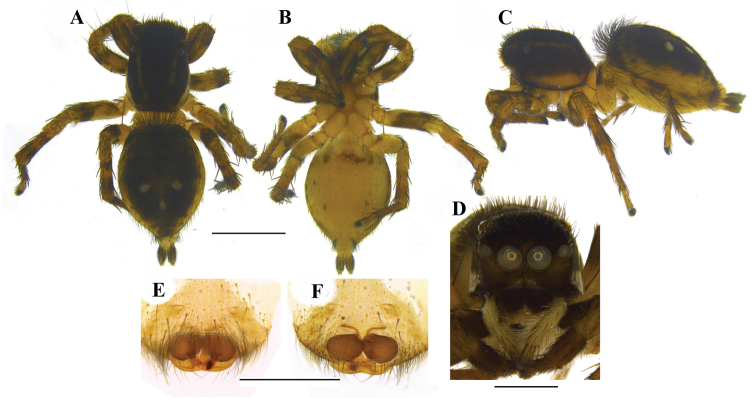
*Stenaelurillus lesserti* Reimoser, 1934. **A** Female habitus, dorsal view **B** Same, ventral view **C** Same, retrolateral view **D** Same, frontal view **E** Epigyne **F** Internal duct system. Scale bars: **A–C** = 2 mm; **D** = 1 mm; **E–F** = 0.5 mm.

**Figure 12.**
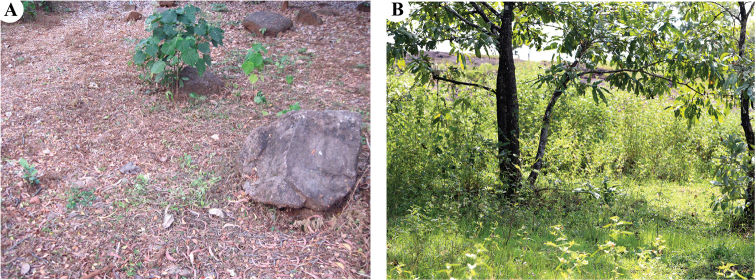
Habitats of *Stenaelurillus* spp. **A** View of the type locality of *Stenaelurillus albus* sp. n. **B** View of the collection site of *Stenaelurillus lesserti* Reimoser, 1934.

## Supplementary Material

XML Treatment for Stenaelurillus

XML Treatment for Stenaelurillus albus

XML Treatment for Stenaelurillus lesserti
